# Increasing carbon availability stimulates growth and secondary metabolites via modulation of phytohormones in winter wheat

**DOI:** 10.1093/jxb/erx008

**Published:** 2017-02-17

**Authors:** Jianbei Huang, Michael Reichelt, Somak Chowdhury, Almuth Hammerbacher, Henrik Hartmann

**Affiliations:** 1 Max Planck Institute for Biogeochemistry, Hans-Knöll-Str. 10, D-07745, Jena, Germany; 2 Max Planck Institute for Chemical Ecology, Hans-Knöll-Str. 8, D-07745, Jena, Germany; 3 Department of Microbiology and Plant Pathology, Forestry and Agricultural Biotechnology Institute, University of Pretoria, Private Bag X20, Pretoria 0028, South Africa

**Keywords:** Abscisic acid, auxin, elevated CO_2_, jasmonic acid, low CO_2_, salicylic acid, secondary metabolites, soluble sugars.

## Abstract

Phytohormones play important roles in plant acclimation to changes in environmental conditions. However, their role in whole-plant regulation of growth and secondary metabolite production under increasing atmospheric CO_2_ concentrations ([CO_2_]) is uncertain but crucially important for understanding plant responses to abiotic stresses. We grew winter wheat (*Triticum aestivum*) under three [CO_2_] (170, 390, and 680 ppm) over 10 weeks, and measured gas exchange, relative growth rate (RGR), soluble sugars, secondary metabolites, and phytohormones including abscisic acid (ABA), auxin (IAA), jasmonic acid (JA), and salicylic acid (SA) at the whole-plant level. Our results show that, at the whole-plant level, RGR positively correlated with IAA but not ABA, and secondary metabolites positively correlated with JA and JA-Ile but not SA. Moreover, soluble sugars positively correlated with IAA and JA but not ABA and SA. We conclude that increasing carbon availability stimulates growth and production of secondary metabolites via up-regulation of auxin and jasmonate levels, probably in response to sugar-mediated signalling. Future low [CO_2_] studies should address the role of reactive oxygen species (ROS) in leaf ABA and SA biosynthesis, and at the transcriptional level should focus on biosynthetic and, in particular, on responsive genes involved in [CO_2_]-induced hormonal signalling pathways.

## Highlight

Auxin and jasmonates are involved in whole-plant regulation of growth and secondary metabolites, whereas abscisic and salicylic acid may be associated with leaf oxidative stress in response to low carbon availability.

## Introduction

Plants capture CO_2_ from the atmosphere and convert it into sugars as essential building blocks for growth and substrates for metabolism (Hartmann and Trumbore, 2016). Atmospheric CO_2_ concentrations ([CO_2_]) has risen from ~170–200 ppm during glacial periods to the current 400 ppm, and are predicted to reach between 430 ppm and 1000 ppm by 2100 (Cubasch *et al.*, 2013). Understanding the mechanisms by which increasing [CO_2_] have influenced whole-plant growth and metabolism in the past will help to unravel mechanisms regulating plant responses to future elevated [CO_2_] but also to reduced carbon availability as may occur during shading, cold, or drought. Phytohormones play an important role in plant acclimation to changing environmental conditions (Peleg and Blumwald, 2011), such as drought (Valluru *et al.*, 2016) and salinity (Albacete *et al.*, 2008). However, our understanding of how phytohormones, such as auxin, abscisic acid (ABA), jasmonic acid (JA), or salicylic acid (SA), are involved in the whole-plant regulation of plant gas exchange, growth, and secondary metabolite (SM) production under changing [CO_2_] is still limited.

ABA plays a role in multiple physiological processes for stress acclimation. For example, ABA is the main regulator of stomatal responses to drought and salinity (Osakabe *et al.*, 2014). Elevated [CO_2_] reduces stomatal conductance and density (Franks *et al.*, 2013), and this is often (Lake and Woodward, 2008; Chater *et al.*, 2015), but not always (Teng *et al.*, 2006; Merilo *et al.*, 2013) associated with increased leaf ABA concentration. Changes in carbohydrate availability have been proposed to be a sensing pathway by which plants may increase ABA biosynthesis at elevated [CO_2_] (Chater *et al.*, 2014). Additional application of glucose can increase ABA biosynthesis (reviewed in León and Sheen, 2003), and the ABA-dependent signalling pathway is essential for sucrose-induced stomatal closure (Kelly *et al.*, 2013). Moreover, the activation of the ABA signalling pathway is involved in inhibition of root growth under osmotic stress (Achard *et al.*, 2006; Rowe *et al.*, 2016).

Auxin (indole-3-acetic acid; IAA) was recognized as an essential plant growth promoter >70 years ago (Enders and Strader, 2015), but much less is known about its role in modulating plant response to abiotic stress (Kazan, 2013). Elevated CO_2_ increased carbohydrate and IAA concentrations and promoted growth in *Arabidopsis thaliana* (Teng *et al.*, 2006; Hachiya *et al.*, 2014) and tomato seedlings (*Solanum lycopersicum*; Wang *et al.*, 2009) but reduced IAA concentrations in roots of sweet pepper (*Capsicum annuum*; Piñero *et al.*, 2014). Niu *et al.* (2011) showed that the auxin-dependent signalling pathway is required for enhancing root development under elevated CO_2_ in Arabidopsis. Moreover, accumulated soluble sugars may be key elicitors for increased IAA production under elevated [CO_2_], as both glucose (Sairanen *et al.*, 2012) and sucrose additions (Lilley *et al.*, 2012) have been shown to stimulate IAA biosynthesis and resulted in higher growth rates in the latter study. In contrast, low [CO_2_] reduces carbohydrate availability (Hartmann *et al.*, 2013, 2015) and limits plant growth (Gerhart and Ward, 2010), but whether IAA regulation is involved in these processes remains uncertain.

JA (Riemann *et al.*, 2015) and SA (Khan *et al.*, 2015) play key roles in regulating plant defence responses to abiotic and biotic stresses. The JA signalling pathway involves the isoleucine (Ile) conjugate of JA (JA-Ile), a phytohormone that activates the transcription of JA-dependent defence genes (Thines *et al.*, 2007). In contrast to JA-Ile, the hydroxylated derivatives of JA, such as 12-hydroxy-JA (12-OH-JA, tuberonic acid) may deactivate JA-dependent defence genes (Miersch *et al.*, 2008; Koo and Howe, 2012). Elevated [CO_2_] has been shown to enhance SA-dependent defence and repress JA-dependent defence (Zavala *et al.*, 2013; Sun *et al.*, 2016), but how plants regulate JA and SA at low [CO_2_] remains uncertain. Furthermore, changes in soluble sugars may regulate production of SMs via modulation of JA and SA. Sucrose (Loreti *et al.*, 2008) and glucose (Guo *et al.*, 2013) play a synergistic role with JA in anthocyanin and glucosinolate biosynthesis, respectively.

Shading and defoliation are common approaches for reducing plant sugar availability, but shading can activate IAA biosynthesis (Casal, 2013) and suppress JA biosynthesis via changes in phytochromes (Ballare, 2014), and defoliation itself is a wounding treatment that can trigger JA biosynthesis with cascading effects on other phytohormones (Erb *et al.*, 2012). Directly manipulating [CO_2_] suffers less from such side effects and allows plants to regulate phytohormones under contrasting carbon (rather than light) availability.

Here, we present an analysis of whole-plant phytohormone dynamics in winter wheat (*Triticum aestivum*) grown along a gradient of atmospheric CO_2_ concentrations (170, 390, and 680 ppm). We investigated correlations between phytohormone concentrations and stomatal conductance, relative growth rate (RGR), and soluble sugar and SM concentrations. In this study, we focused on ABA, IAA, SA, and JA as well as its derivatives JA-Ile and 12-OH-JA, although we are aware that other phytohormones such as cytokinin and ethylene also play important roles in the regulation of growth. Based on knowledge about hormonal regulation of growth and secondary metabolite production at the tissue/organ level, we explore whether the following relationships hold true at the whole-plant level: with increasing [CO_2_], plants (i) reduce stomatal conductance via increasing ABA; (ii) promote growth via increasing IAA; (iii) increase biosynthesis of SMs via increasing JA, JA-Ile, and SA; and (iv) show higher carbohydrate concentrations that, in turn, induce the biosynthesis of phytohormones.

## Materials and methods

### Plant material

We used a cultivar of winter wheat (*Triticum aestivum* cv. Genius) adapted to Central Europe. On 7 January 2015, seeds were germinated on plates filled with sand and watered every day. After 6 d, we transplanted 10–12 seedlings with similar height into each pot (11 cm diameter, 24 cm height) pre-filled with quartz sand. Six pots were randomly placed in each growth chamber. All pots were irrigated with a continuous through-flow of a modified Hoagland solution (Hoagland and Arnon, 1950).

### Growth chambers and treatments

Plants were grown in glass chambers (75 cm long×45 cm wide×80 cm high) flushed continuously with air at a flow rate of 14 l min^−1^ (for more details, see Hartmann *et al.*, 2013). Concentrations of the different CO_2_ treatments were produced by first scrubbing all CO_2_ from incoming air using a molecular sieve, and then injecting pure CO_2_ (Schnyder, 1992). The [CO_2_] of incoming air for the three [CO_2_] treatments was measured with a Vaisala^®^ (GMP 343) at intervals of 10 min. A micro-logger (Campbell^®^ CR1000) compared these concentrations against pre-set values (170, 390, and 680 ppm) and adjusted them accordingly via mass flow controllers. For each [CO_2_] treatment, three chambers were used to grow plants and one chamber was used to monitor [CO_2_] reference levels.

In each chamber, we monitored air temperature and photosynthetic photon flux density (PPFD) continuously (for more details, see Hartmann *et al.*, 2013). During the day, average temperature increased from ~12 °C at 06:30 h (local time) to ~20–24 °C and then decreased to ~16.5 °C at 22:00 h. Plants were grown in a light/dark regime of 16/8 h using supplemental greenhouse lamps. The average PPFD from 23 January 2015 to 20 March 2015 was 7.79 ± 0.92 mol m^−2^ d^−1^.

### Destructive harvesting

To rule out potential effects of plant development on hormone levels, we harvested plants in different treatments independently of calendar dates and when three, six, and eight leaf sheaths were completely developed, denoted as 3L, 6L, and 8L periods, respectively. The experiment was only conducted during the vegetative growth period (seedling growth and tillering stage). Plants grown in 390 ppm and 680 ppm [CO_2_] chambers were sampled 3, 7, and 9 weeks after transplanting, whereas plants grown in low [CO_2_] chambers were sampled 3.5, 8, and 10.5 weeks after transplanting. Harvests were always conducted between 16:00 h and 21:00 h to minimize light and temperature effects on hormones and metabolites. For each harvest, we removed one pot from each chamber. Plants were separated into leaves, stems, and roots. Leaf area was determined with a Li 3100A area meter (Li-Cor, Bad Homburg, Germany). All fresh tissues were weighed and frozen in liquid nitrogen and later transferred to a −80 °C freezer. Around 75% of the biomass was freeze-dried, weighed, and ground to fine powder using a ball mill (Retsch^®^ MM400, Haan, Germany) and finally stored at −20 °C until further analysis. The rest of the samples were ground with liquid nitrogen using a mortar and pestle, and stored at −80 °C until analysis.

### Whole-plant gas exchange

A Picarro^®^ 2101-i (precision 0.01–0.4%, Picarro Inc., Santa Clara, CA, USA) was used to measure the [CO_2_] and [H_2_O] of air entering and leaving the growth chambers. The air coming from the 12 chambers and the reference air were measured sequentially at intervals of 6 min 40 s; the cycle was controlled by a micro-logger (Campbell^®^ CR1000) connected to a custom-built valve switching unit, completing a whole cycle within 2 h. Transition periods after valve switching were excluded from analysis.

We assumed whole-plant gas exchange to be constant within the 2 h cycle. The instantaneous whole-plant assimilation (*A*) and transpiration (*E*) at hour *j* was calculated as:

$${\left[{\text{CO}}_{2}\;\text{or }{\text{H}}_{2}\text{O}\right]}_{j}\;\left(µmol\;s^{-1}\right)=\;\frac{\begin{array}{l} {\left[{\text{CO}}_{2}\;\text{or }{\text{H}}_{2}\text{O}\right]}_{\text{non}-\text{plant}}–\\ \;{\left[{\text{CO}}_{2}\;\text{or }{\text{H}}_{2}\text{O}\right]}_{\text{plant}}\; \end{array}}{M}\left(\text{µmol }{\text{mol}}^{-1}\right)$$

$$\;\times \frac{\text{VFR}\left(\text{l }{\text{min}}^{-1}\right)}{\;22.4\;\left(\text{l }{\text{mol}}^{-1}\right)\times 60\;\text{s}\;}$$ (1)

where [CO_2_ or H_2_O]_non-plant_ and [CO_2_ or H_2_O]_plant_ is the [CO_2_ or H_2_O] of outgoing air from the reference chambers without plants and from chambers with plants, respectively. *M* represented the number of plants in the chamber, and VFR was the volumetric flow rate of air going through the chamber (14 l min^−1^). The value of 22.4 l mol^−1^ is the molar volume of gas under normal conditions. Canopy conductance was then estimated as previously described in McDowell *et al.* (2008):

$$G_{S}=\frac{E}{\text{VPD}}\;$$ (2)

where VPD (kPa) is vapour pressure deficit. Note that the relative humidity of incoming air was very low (<2%) therefore VPD is close to the saturation vapour pressure at chamber temperature. Leaf area-based (i.e. specific) net assimilation, transpiration, and stomatal conductance (*G*_s_) were then calculated by dividing instantaneous whole-plant exchange by leaf area (m^2^). To obtain a robust estimate of current whole-plant gas exchange, we averaged whole-plant gas exchange over the last few days prior to biomass sampling.

### Analysis of soluble sugars

Concentrations of glucose, sucrose, and fructose were measured using the method of Raessler *et al.* (2010). Briefly, we added 1 ml (0.5 ml for small samples) of sterilized water to 50 mg (10 mg for small samples) of ground sample. The mixture was vortexed, incubated for 10 min at 65 °C, and then centrifuged for 10 min at 12 000 *g*. The supernatant was carefully collected and stored on ice, and the pellet was re-extracted twice. The supernatants were pooled and diluted at a ratio of 1:20 (1:8 for small samples) and stored at −20 °C before measurements. Sucrose, glucose, and fructose were determined by HPLC coupled with pulsed amperometric detection (HPLC-PAD), on a Dionex^®^ ICS 3000 ion chromatography system equipped with an autosampler (Thermo Fisher GmbH, Idstein, Germany).

### Analysis of SMs

A 500 µl aliquot (300 µl for small samples) of 95% methanol was added to 50 mg (30 mg for small samples) of freeze-dried tissues. The mixture was bead-beaten for 40 s at 6.0 m s^−1^ with a FastPrep Instrument (MP Biomedicals, Santa Ana, CA, USA), vortexed for 5 min, and then centrifuged at 13 000 *g* for 5 min. The supernatant was collected and the pellet was re-extracted. The supernatants were pooled and stored at 4 °C. Identification and quantification of SMs were achieved by HPLC coupled with MS and a UV Detector. Phenolic compounds were separated on a Nucleodur Sphinx RP18ec column (250 × 4.6 mm, particle size 5 µm, Macherey Nagel, Dueren, Germany) with two mobile phases 0.2% (v/v) formic acid (A) and acetonitrile (B) using the following elution profile: 0–28 min, 5–61% B in A; 28–30 min 100% B; and 30–35 min 5% B. Flow was diverted in a ratio of 4:1 before entering the mass spectrometer electrospray chamber. For identification, ESI-MS was operated at a negative mode scanning *m/z* between 50 and 1600 with an optimal target mass of 400 *m/z*. The MS conditions were: skimmer voltage, 60 V; capillary voltage, 4200 V; nebulizer pressure, 35 psi; drying gas, 11 l min^−1^; gas temperature, 330 °C; capillary exit potential, −121 V. For quantification, the UV wavelengths 240, 260, 280, and 330 nm were monitored. Compounds of leaves and stems were identified by comparing the fragmentation patterns with previously reported wheat phenolic profiles (Moheb *et al.*, 2011; Wojakowska *et al.*, 2013). Root compounds were identified based on profiles of benzoxazinoids in grasses (Wouters *et al.*, 2014). All compounds were quantified by external standards (for more details, see Huang *et al.*, 2016).

### Quantification of hormones

Concentrations of ABA, IAA, SA, and jasmonates, comprising JA, JA-Ile, and 12-OH-JA, were determined using the method of Vadassery *et al.* (2012) with modifications. Briefly, 250 mg of fresh samples were extracted with 1 ml of methanol containing 40 ng of D_6_-ABA (Santa Cruz Biotechnology, Santa Cruz, CA, USA), 40 ng of D_5_-IAA (Olchemin, Olomouc, Czech Republic), 40 ng of D_4_-SA (Sigma-Aldrich), 40 ng of D_6_-JA (HPC Standards GmbH, Cunnersdorf, Germany), and 8 ng of JA-[^13^C_6_]Ile conjugate as internal standards. JA-[^13^C_6_]Ile was synthesized using [^13^C_6_]Ile (Sigma-Aldrich) according to Kramell *et al.* (1988). The mixture was vortexed for 10 min and then centrifuged at 13 000 *g* for 10 min. A 800 µl aliquot of the supernatant was then collected and transferred into a 5 ml 96-well plate. The pellet was re-extracted with 500 µl of methanol using the same procedure, and 500 ml of supernatant was collected, pooled, and stored at −20 °C.

 Hormone detection and quantification was accomplished with an Agilent 1260 HPLC system (Agilent Technologies, Santa Clara, CA, USA) coupled to an API 5000 tandem mass spectrometer (Applied Biosystems, Foster City, CA, USA) equipped with a Turbospray ion source. Hormones were separated on a Zorbax Eclipse XDB-C18 HPLC column (1.8 µm, 50 × 4.6 mm; Agilent) at 25 °C, with two mobile phases consisting of 0.05% formic acid in water (solvent A) and acetonitrile (solvent B), at a flow rate of 1.1 ml min^–1^ using the following elution profile: 0–0.5 min, 10% B; 0.5–4.0 min, linear gradient from 10% to 90% B; 4.0–4.02 min, linear gradient from 90% to 100% B; 4.02-4.50 min, 100% B, 4.50-4.51 min, linear gradient from 100% to 10% B; and 4.51–7.00 min, 10% B. The parent ion and their fragments of jasmonates, SA, and ABA were analysed in negative mode by multiple reaction monitoring (MRM) (for more details, see Vadassery *et al.*, 2012). IAA was analysed in the positive ionization mode in a separate chromatographic analysis (same LC conditions as above for other phytohormones) with the following conditions: analyte parent ion→product ion: *m/z* 176→130 for IAA; *m/z* 181→134+*m/z* 181→133 for D_5_-IAA. Collision energy (CE) was 19 V; declustering potential (DP) was 31 V. Q1 and Q3 quadrupoles were both maintained at unit resolution. Mass data were collected and processed using analyst 1.6 software (Applied Biosystems). Linearity in ionization efficiencies was confirmed by analysing serial dilutions of a standard mixture. The concentrations of ABA, IAA, SA, JA, and JA-Ile were determined relative to the corresponding internal standard. The concentration of OH-JA was determined relative to D_6_-JA by analysing a mixture of OH-JA and D_6_-JA at the same concentration. OH-JA was synthesized as described in Nakamura *et al.* (2011) and was kindly provided by Wilhelm Boland (MPI for Chemical Ecology, Jena, Germany).

### Data analysis

Each growth chamber was treated as a biological replicate (*n*=3). We determined homogeneity of variances with the Levene test and log-transformed data when variance was not homoscedastic. Tukey’s HSD (*P*<0.05) was used to detect significant differences between treatments. Weighted phytohormone concentrations were calculated by multiplying tissue-specific concentrations by tissue biomass and dividing their sum by whole-plant mass, as for weighted soluble sugars and SM concentrations. Phytohormones were reported on a fresh weight basis; therefore, to ensure consistency of units, the concentrations of soluble sugars and SMs were converted to fresh weight. We assessed the Pearson’s correlation of phytohormones with gas exchange, RGR, soluble sugars, and SMs. All statistical analysis was conducted in R, version 3.23 (R Development Core Team, 2014).

## Results

### Gas exchange rate

**Fig. 1.**
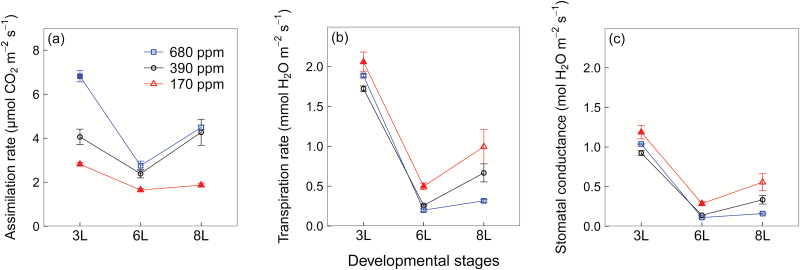
Leaf area-based net assimilation rate (a), transpiration rate (b), and stomatal conductance of winter wheat (*Triticum aestivum*) for the three [CO_2_] treatments (squares, 680 ppm; circles, 390 ppm; triangles, 170 ppm). Values are the mean ±SE of three individual chambers. Filled symbols of 680 ppm and 170 ppm [CO_2_] treatments indicate significant differences compared with 390 ppm [CO_2_] treatment (*P*<0.05, Tukey’s HSD). We harvested plants after emergence of three, six, and eight leaf sheaths, denoted by 3L, 6L, and 8L, respectively. (This figure is available in colour at *JXB* online.)

Plants grown at 170 ppm [CO_2_] exhibited lower assimilation rates but higher transpiration rates and higher stomatal conductance compared with plants grown at 390 ppm and 680 ppm [CO_2_] (Fig. 1a–c). The difference between the two higher [CO_2_] treatments, however, varied with developmental stages. At 3L, assimilation rates increased significantly at 680 ppm [CO_2_] compared with 390 ppm [CO_2_], while at 6L and 8L the increase disappeared (Fig. 1a). At 3L and 6L, transpiration rates and stomatal conductance remained relatively constant between 390 ppm and 680 ppm [CO_2_], while at 8L they were higher at 680 ppm than at 390 ppm [CO_2_] (Fig. 1b, c).

### RGR, soluble sugars, and secondary metabolites

**Fig. 2.**
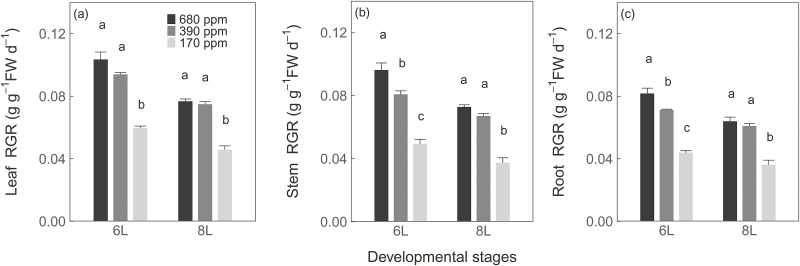
Relative growth rate (RGR) of leaves (a), stems (b), and roots (c) of winter wheat (*Triticum aestivum*) for the three [CO_2_] treatments. Values are the mean (g g^−1^ FW d^−1^) ±SE of three individual chambers. Significant differences between [CO_2_] treatments are indicated by different letters (*P*<0.05, Tukey’s HSD). We harvested plants after emergence of three, six, and eight leaf sheaths, and RGR between three and six leaf sheaths and between six and eight leaf sheaths are denoted by 6L and 8L, respectively.

Similar to assimilation, plants grown at 170 ppm [CO_2_] exhibited a lower RGR of all tissues than plants grown at 390 ppm [CO_2_], across developmental stages (Fig. 2a–c). At 6L, RGR was higher in plants grown at 680 ppm [CO_2_] than at 390 ppm [CO_2_], but at 8L, plants grown at 390 ppm and 680 ppm [CO_2_] exhibited a similar RGR of all tissues (Fig. 2a–c).

**Fig. 3.**
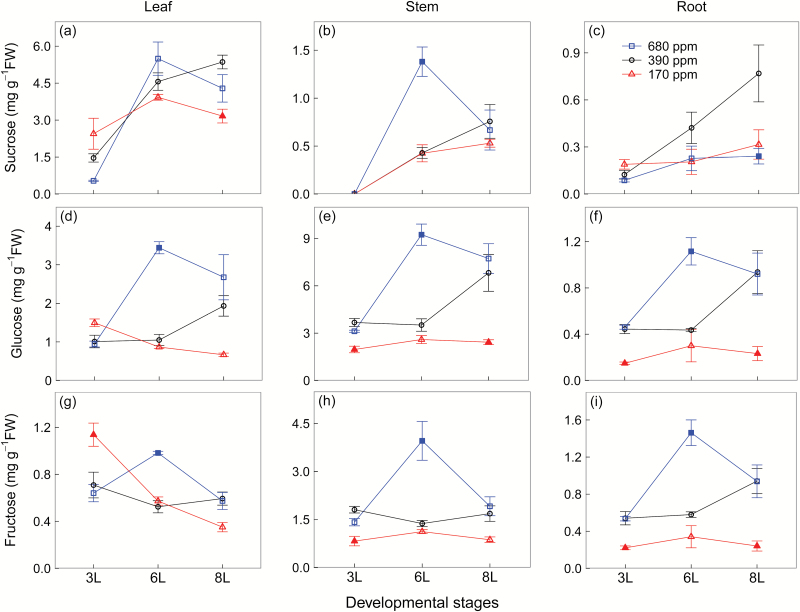
Sucrose (a–c), glucose (d–f), and fructose (g–i) concentrations of winter wheat (*Triticum aestivum*) for the three [CO_2_] treatments (squares, 680 ppm; circles, 390 ppm; triangles, 170 ppm). Values are the mean (mg g^−1^ FW) ±SE of three individual chambers. Filled symbols of 680 ppm and 170 ppm [CO_2_] treatments indicate significant differences compared with 390 ppm [CO_2_] treatment (*P*<0.05, Tukey’s HSD). Note that the concentrations are expressed on a fresh weight basis and at different scales. We harvested plants after emergence of three, six, and eight leaf sheaths, denoted by 3L, 6L, and 8L, respectively. (This figure is available in colour at *JXB* online.)

The [CO_2_] response of soluble sugars varied with developmental stages and tissues. At 3L, all soluble sugar concentrations slightly increased at 170 ppm [CO_2_] in leaves, but glucose and fructose concentrations significantly decreased in stems and roots, compared with 390 ppm [CO_2_] (Fig. 3a–i). At 6L, there was no significant difference in soluble sugars across tissues between 170 ppm and 390 ppm [CO_2_], but glucose and fructose concentrations significantly increased at 680 ppm [CO_2_] in all tissues compared with 390 ppm [CO_2_] (Fig. 3a–i). Interestingly, at 6L, we did not observe large differences in leaf sucrose concentration between the two higher [CO_2_] treatments. From 6L to 8L, while soluble sugars of all tissues showed a declining trend at 680 ppm [CO_2_], they accumulated at 390 ppm [CO_2_] but slightly decreased at 170 ppm [CO_2_] (Fig. 3a–i). Sucrose concentrations were much higher in leaves than in stems and roots, whereas glucose and fructose concentrations were generally higher in stems (Fig. 3a–i).

At 3L, plants grown at contrasting [CO_2_] showed similar SM concentrations in leaves and roots. However, at 6L and 8L, leaf SM concentrations decreased with declining [CO_2_], but the decrease was greater at 170 ppm (compared with 390 ppm) than at 390 ppm (compared with 680 ppm). In contrast, at 6L and 8L, root SM concentrations remained unchanged between 390 ppm and 680 ppm [CO_2_] treatments, but they were lower at 170 ppm than at 390 ppm and 680 ppm [CO_2_], although not statistically significant at 8L (Table 1).

**Table 1.** Secondary metabolite concentrations in leaves, stems, and roots of winter wheat (*Triticum aestivum*) for the three [CO_2_] treatments: 680 ppm [CO_2_], 390 ppm [CO_2_], and 170 ppm [CO_2_] Values are mean (µg g^−1^ FW) ±SD of three individual chambers. Note that the concentrations are expressed on a fresh weight basis.

Development	CO_2_ ppm	Leaf					Stem	Root		
		Ferulic acid	Luteolin	Apigenin	Chrysoeriol	Tricin	Ferulic acid	Putrescine	DIMBOA-Glc	HDMBOA- Glc
3L	680	88.8 (6.1) a	59.8 (9.8) a	155.9 (15.5) a	43.5 (3.7) a	47.0 (3.9) a	55.9 (2.2) a	16.0 (4.3) a	46.3 (10.7) a	47.9 (6.5) a
	390	92.5 (10.2) a	51.3 (10.6)ab	163.1 (13.7) a	45.1 (4.5) a	48.3 (3.0) a	53.0 (4.7) a	20.3 (4.9) a	48.9 (6.2) a	44.5 (7.6) a
	170	87.9 (1.9) a	29.4 (5.5) b	134.2 (17.4) a	35.4 (4.8) a	43.3 (4.2) a	42.5 (3.7) b	18.2 (4.2) a	61.5 (24.4) a	58.0 (3.4) a
6L	680	57.9 (2.8) a	56.1 (4.7) a	193.8 (4.5) a	45.1 (2.5) a	70.7 (6.9) a	34.5 (0.5) a	32.1 (6.6) ab	59.4 (8.9) a	31.2 (2.4) a
	390	53.9 (6.3) ab	36.7 (3.2) b	164.4 (7.8) b	39.1 (3.0) b	50.2 (2.4) b	30.5 (2.5) b	45.6 (6.8) a	65.7 (13.4) a	27.8 (2.3) ab
	170	45.6 (2.6) b	11.8 (2.6) c	112.0 (11.3) c	25.4 (1.2) c	38.4 (4.0) b	29.0 (0.3) b	20.6 (4.3) b	44.0 (3.6) a	22.8 (2.3) b
8L	680	40.6 (3.4) a	39.8 (2.9) a	179.2 (8.8) a	39.5 (2.6) a	67.5 (7.4) a	29.2 (1.7) a	48.4 (5.6) a	56.1 (6.9) a	26.1 (4.3) a
	390	43.7 (1.5) a	26.9 (1.0) b	158.0 (10.7) a	33.4 (1.7) a	53.0 (7.4) b	31.4 (0.8) a	54.5 (12.9) a	57.4 (5.3) a	22.8 (1.3) a
	170	37.9 (4.7) a	6.1 (2.1) c	101.5 (10.7) b	17.7 (3.2) b	35.9 (2.5) c	23.6 (5.1) a	32.5 (10.9) a	44.8 (7.6) a	21.7 (7.4) a

Different letters indicate significant differences between [CO_2_] treatments (*P*<0.05, Tukey’s HSD).

We harvested plants after emergence of three, six, and eight leaf sheaths, denoted by 3L, 6L, and 8L, respectively.

DIMBOA-Glc, 2-(2,4-dihydroxy-7-methoxy-1,4-benzoxazin-3-one)-β-d-glucopyranose; HDMBOA-Glc, 2-(2-hydroxy-4,7-dimethoxy-1,4- benzoxazin-3-one)-β-d-glucopyranose. See also supporting information from Huang *et al*. (2016).

### Hormonal profiling

**Fig. 4.**
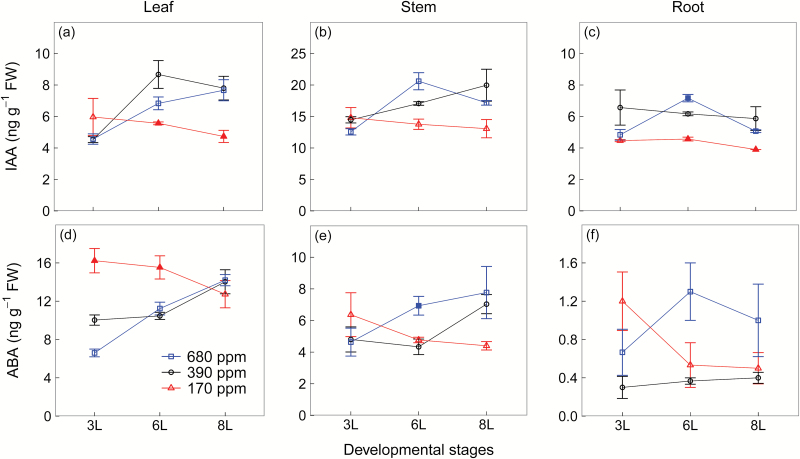
Auxin (IAA) (a–c) and abscisic acid (ABA) concentrations of winter wheat (*Triticum aestivum*) for the three [CO_2_] treatments (squares, 680 ppm; circles, 390 ppm; triangles, 170 ppm). Values are the mean (ng g^−1^ FW) ±SE of three individual chambers. Filled symbols of 680 ppm and 170 ppm [CO_2_] treatments indicate significant differences compared with 390 ppm [CO_2_] treatment (*P*<0.05, Tukey’s HSD). Note that the concentrations are expressed on a fresh weight basis and at different scales. We harvested plants after emergence of three, six, and eight leaf sheaths, denoted by 3L, 6L, and 8L, respectively. (This figure is available in colour at *JXB* online.)

At 6L and 8L, IAA concentrations decreased at 170 ppm [CO_2_] in all tissues compared with 390 ppm and 680 ppm [CO_2_], but the difference between the two higher [CO_2_] treatments varied with developmental stages and tissues (Fig. 4a–c). IAA concentrations in stems were generally twice more than those in leaves and roots (Fig. 4a–c). In contrast to IAA, at 3L and 6L, ABA concentrations increased at 170 ppm [CO_2_] in leaves but not in stems and roots, compared with 390 ppm and 680 ppm [CO_2_] (Fig. 4d, e). From 3L to 8L, a consistent decrease in leaf ABA concentrations was observed at 170 ppm [CO_2_], while the opposite was the case for 390 ppm and 680 ppm [CO_2_] treatments (Fig. 4d). Note that ABA concentrations were much lower in roots than in leaves and stems (Fig. 4d, e).

**Fig. 5.**
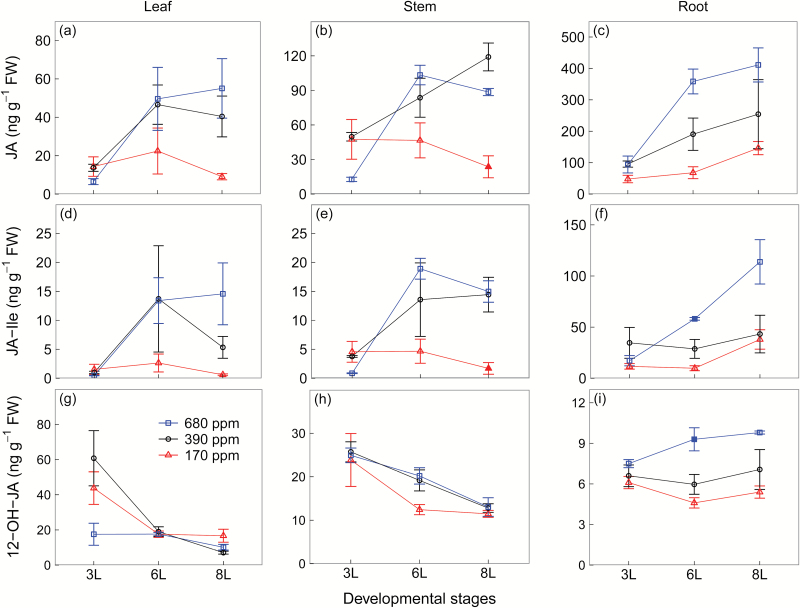
Jasmonic acid (JA) (a–c), the isoleucine (Ile) conjugate of JA (JA-Ile) (d–f), and 12-hydroxy-JA (12-OH-JA) (g–i) concentrations of winter wheat (*Triticum aestivum*) for the three [CO_2_] treatments (squares, 680 ppm; circles, 390 ppm; triangles, 170 ppm). Values are the mean (ng g^−1^ FW) ±SE of three individual chambers. Filled symbols of 680 ppm and 170 ppm [CO_2_] treatments indicate significant differences compared with 390 ppm [CO_2_] treatment (*P*<0.05, Tukey’s HSD). Note that the concentrations are expressed on a fresh weight basis and at different scales. We harvested plants after emergence of three, six, and eight leaf sheaths, denoted by 3L, 6L, and 8L, respectively. (This figure is available in colour at *JXB* online.)

Similar to IAA, at 6L and 8L, JA and JA-Ile concentrations were much lower in plants grown at 170 ppm than at 390 ppm and 680 ppm [CO_2_], but at 6L there was no difference in leaves and stems between these two higher [CO_2_] treatments (Fig. 5a–e). Plants grown at contrasting [CO_2_] exhibited relatively similar 12-OH-JA concentrations in leaves and stems (Fig. 5g–i). Note that leaves and stems had lower concentrations of JA and JA-Ile but higher concentrations of 12-OH-JA compared with roots, in particular at 390 ppm and 680 ppm [CO_2_] (Fig. 5a–i).

**Fig. 6.**
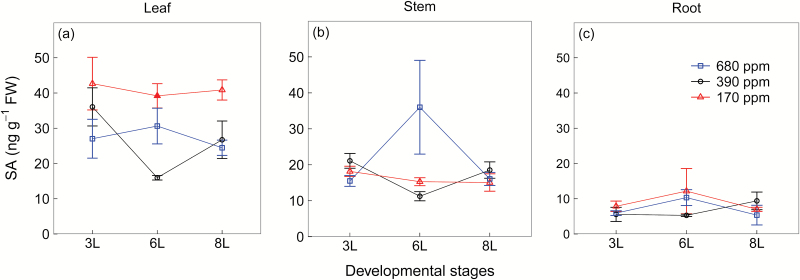
Salicylic acid (SA) (a–c) concentrations of winter wheat (*Triticum aestivum*) for the three [CO_2_] treatments (squares, 680 ppm; circles, 390 ppm; triangles, 170 ppm). Values are the mean (ng g^−1^ FW) ±SE of three individual chambers. Filled symbols of 680 ppm and 170 ppm [CO_2_] treatments indicate significant differences compared with 390 ppm [CO_2_] treatment (*P*<0.05, Tukey’s HSD). Note that the concentrations are expressed on a fresh weight basis. We harvested plants after emergence of three, six, and eight leaf sheaths, denoted by 3L, 6L, and 8L, respectively. (This figure is available in colour at *JXB* online.)

In contrast, plants grown at 170 ppm [CO_2_] had higher leaf SA concentrations than at 390 ppm and 680 ppm [CO_2_] (Fig. 6a). However, at 3L and 8L, there were no differences between the two higher [CO_2_] treatments, and only at 6L were leaf SA concentrations lower at 390 ppm [CO_2_] than at 680 ppm [CO_2_] (Fig. 6a). SA concentrations remained relatively constant in stems and roots across [CO_2_] treatments (Fig. 6b, c). Note that SA concentrations were relatively lower in stems and roots than in leaves (Fig. 6a–c).

### Correlations of phytohormones to RGR, stomatal conductance, SMs, and soluble sugars

**Fig. 7.**
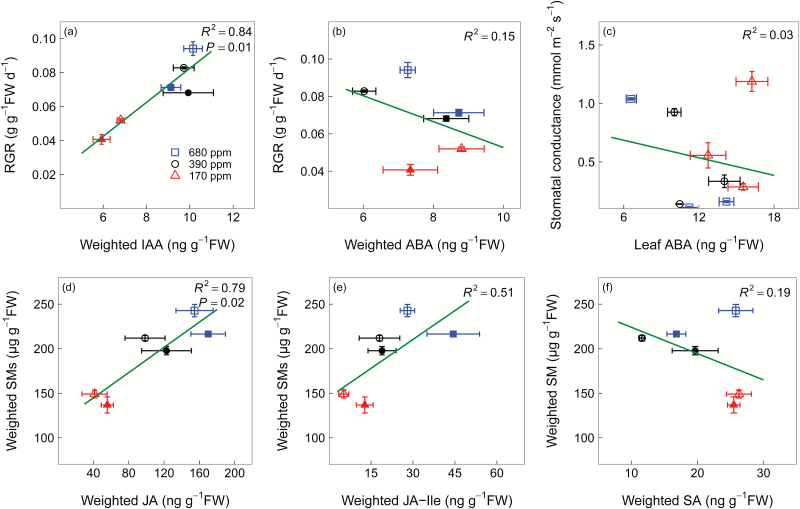
Pearson’s correlations of whole-plant relative growth rate (RGR) to weighted auxin (IAA) (a) and abscisic acid (ABA) (b) concentrations, between leaf area-based stomatal conductance and leaf ABA concentrations (c), and Pearson’s correlations of weighted secondary metabolite (SM) concentration to jasmonic acid (JA) (d), isoleucine (Ile) conjugate of JA (e), and salicylic acid (SA) concentrations (f) in winter wheat (*Triticum aestivum*). The three [CO_2_] treatments: squares, 680 ppm; circles, 390 ppm; triangles, 170 ppm. We harvested plants after emergence of three, six, and eight leaf sheaths, denoted by 3L, 6L, and 8L, respectively. However, before 3L, RGR and SM concentrations were also affected by seed storage, independent of [CO_2_]; therefore, we only show RGR and SM concentrations of 6L and 8L, where open symbols represent 6L and filled symbols represent 8L. In contrast, we show stomatal conductance from 3L, 6L, and 8L, and all values are shown by open symbols. Values are the mean ±SE of three individual chambers. Note that the concentrations are expressed on a fresh weight basis and at different scales. (This figure is available in colour at *JXB* online.)

**Fig. 8.**
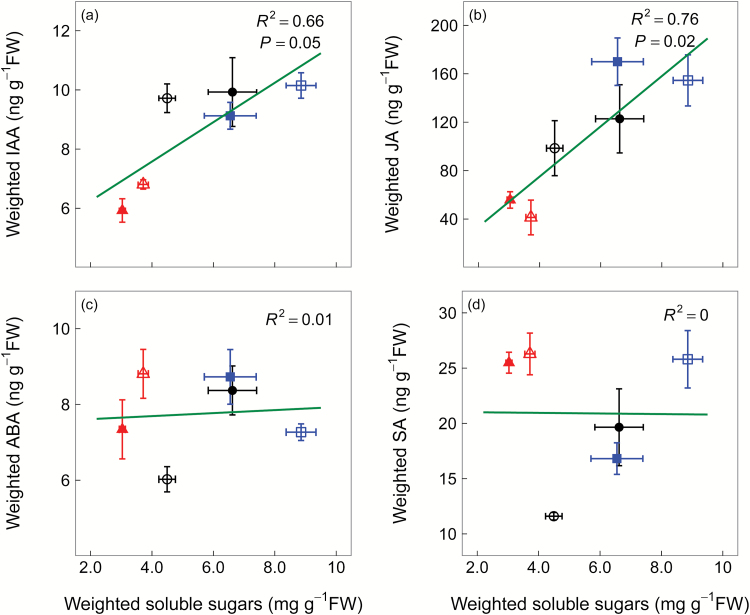
Pearson’s correlations of weighted soluble sugar concentration (mg g^−1^ FW) to weighted auxin (IAA) (a), jasmonic acid (JA) (b), abscisic acid (ABA) (c), and salicylic acid (SA) (d) concentrations (ng g^−1^ FW) in winter wheat (*Triticum aestivum*). The three [CO_2_] treatments: squares, 680 ppm; circles, 390 ppm; triangles, 170 ppm. We harvested plants after emergence of three, six, and eight leaf sheaths, denoted by 3L, 6L, and 8L, respectively. However, before 3L, soluble sugar and SM concentrations were also affected by seed storage, independent of [CO_2_]; therefore, we only show data of 6L and 8L, where open symbols represent 6L and filled symbols represent 8L. Values are the mean ±SE of three individual chambers. Note that the concentrations are expressed on a fresh weight basis and at different scales. (This figure is available in colour at *JXB* online.)

As expected, the decline in whole-plant RGR with decreasing [CO_2_] was positively correlated with weighted IAA concentrations (*R*^2^=0.84, *P*=0.01) (Fig. 7a). In contrast, RGR was negatively correlated with weighted ABA concentrations (*R*^2^=0.15), but not statistically significantly (Fig. 7b). There was no correlation between stomatal conductance and leaf ABA concentrations (Fig. 7c). Weighted SM concentrations were also positively correlated with weighted JA concentrations (*R*^2^=0.79, *P*=0.02) as well as concentrations of its bioactive derivative JA-Ile (*R*^2^=0.51, *P*=0.11), but not with weighted SA concentrations (Fig. 7d, f). We also found a significant positive correlation between weighted soluble sugar concentrations and weighted IAA and JA concentrations, but not weighted ABA and SA concentrations (Fig. 8a–d).

## Discussion

**Fig. 9.**
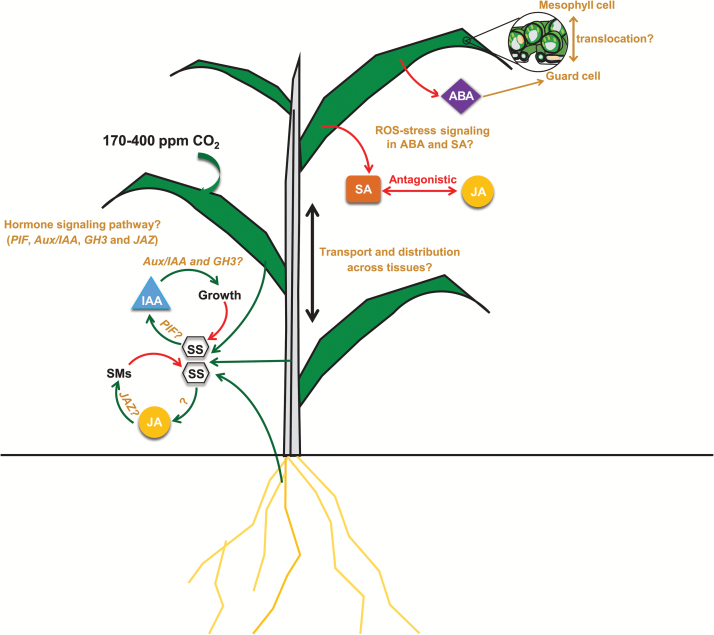
Conceptual model and research needs of whole-plant hormonal regulation in response to changing [CO_2_], derived from our experimental results and published literature. The leaf cross-section figure was modified from an original figure provided by Zephyris (Richard Wheeler) and Wikipedia. Increased [CO_2_] stimulates photosynthesis and results in accumulation of soluble sugars at the whole-plant level which may elicit a cascade of downstream IAA and JA regulation of plant growth and defence, respectively. Increasing [CO_2_] may dissipate the reducing power from the photosynthetic electron transfer chain and reduce photorespiration, which theoretically reduce ROS production (Galvez-Valdivieso *et al.*, 2009). Low [CO_2_]-induced ROS accumulation may trigger ABA (Mittler and Blumwald, 2015) and SA biosynthesis (Herrera-Vásquez *et al.*, 2015), which in turn promote ROS accumulation or scavenging. Low [CO_2_] could increase translocation of ABA from mesophyll chloroplast to guard cells and, as reported by Chater *et al.* (2015), the increase in guard cell ABA is sufficient to reduce stomatal conductance and density. Up-regulation of SA signalling may be associated with suppression of JA signalling (Zavala *et al.*, 2013; Sun *et al.*, 2016). Interactive signalling pathways via sugars for growth regulation and synthesis of secondary metabolites but also transport and distribution of phytohormones across tissues are still uncertain. Soluble sugars may stimulate IAA via the *PIF* family of transcription factors (Lilley *et al.*, 2012; Sairanen *et al.*, 2012). Furthermore, IAA may induce the *GH3* family which in turn catalyses the conjugation of IAA to amino acids (Sauer *et al.*, 2013). IAA and JA may stimulate growth and SM production via degradation of the repressor *Aux/IAA* (Enders and Strader, 2015) and *JAZ* (Riemann *et al.*, 2015), respectively. ABA, abscisic acid; IAA, auxin; JA, jasmonic acid; *JAZ*, jasmonate ZIM-domain protein; *PIF*, phytochrome-interacting factor; ROS, reactive oxygen species; SA, salicylic acid; SMs, secondary metabolites; SS, soluble sugars. Green arrows indicate positive, red negative, and brown uncertain.

Manipulating whole-plant carbon availability along a gradient of [CO_2_] combined with whole-plant hormonal analysis allowed unravelling of the mechanisms by which plants cope with abiotic stresses. As summarized in Fig. 9, our study revealed that low [CO_2_] increased ABA and SA concentrations in leaves, probably in order to cope with potential oxidative stress from excess light excitation energy. With increasing C availability, wheat plants increased growth and SM production via increases in IAA and JA (JA-Ile) levels and probably triggered by sugar signalling pathways.

### Stomatal regulation

Transpiration rates and stomatal conductance were higher at low [CO_2_] than at ambient and elevated [CO_2_], and this difference increased over time. However, contrary to our expectation, stomatal conductance was not associated with leaf ABA concentrations under contrasting [CO_2_]. Although it is well established that ABA can induce stomatal closure (Osakabe *et al.*, 2014), a lack of response to changes in bulk ABA cannot rule out ABA-dependent stomatal regulation, as mutants with only 10% of wild-type ABA concentrations showed stomatal closure under elevated [CO_2_] (Merilo *et al.*, 2013). A recent study of ABA-deficient plants suggested that stomatal regulation under elevated [CO_2_] requires increases in ABA in both stomatal precursor cells and guard cells, rather than in bulk ABA (Chater *et al.*, 2015). Furthermore, increasing leaf ABA concentration between 3L and 8L at elevated [CO_2_] may be due to a down-regulation of stomatal density under elevated [CO_2_] (Chater *et al.*, 2014).

### Growth regulation

RGR increased in all tissues with increasing [CO_2_] and were, at the whole-plant level, positively correlated with IAA but not with ABA, suggesting that IAA may be involved in growth regulation under low C availability. Growth modulation via IAA has also been observed under elevated [CO_2_] in Arabidopsis (Hachiya *et al.*, 2014) and in tomato seedlings (Wang *et al.*, 2009), and during drought and low temperatures in rice (*Oryza sativa*) (Zhang *et al.*, 2009; Du *et al.*, 2013).

While leaves, stems, and roots showed similar RGR, IAA concentrations in stems were more than twice as high as in leaves and roots. IAA is synthesized mainly in the shoot apex and then transported to roots (Ljung *et al.*, 2001) so high IAA concentrations in stems may be an inactive transitory pool. Moreover, the basipetal transport of IAA from shoot to roots remained relatively constant even under low C availability, as the ratio of stem to root IAA concentrations remained relatively constant across [CO_2_] treatments. In addition, at 8L, when RGR and soluble sugar concentrations at elevated [CO_2_] declined in all tissues to ambient [CO_2_] levels, we also observed a decrease in stem IAA concentrations. This decrease highlights the potential role of IAA in photosynthetic acclimation (Xu *et al.*, 2015), especially under elevated [CO_2_].

We observed a weak relationship between RGR and ABA, and inconsistent temporal changes across tissues, suggesting that bulk ABA is not a main regulator for tissue growth. However, the ratio of leaf to stem ABA was, throughout the experiment, consistently higher at low than at ambient [CO_2_], possibly because ABA is involved in photosynthetic acclimation to low [CO_2_]. However, our data set cannot provide deeper insights into this mechanism, and assessments of other parameters, such as enhanced production of reactive oxygen species (ROS) from excess excitation energy at low [CO_2_] (Galvez-Valdivieso *et al.*, 2009), are required to elucidate the role of ABA in stress mitigation (Mittler and Blumwald, 2015).

### Regulation of secondary metabolite synthesis

Low [CO_2_] reduced leaf flavonoids and root putrescine-based compounds as well as root benzoxazinoid derivatives, and this response was strongly associated with reduced JA and its bioactive derivative JA-Ile, but not with its inactive derivative 12-OH-JA. Jasmonates are involved in the biosynthesis of a wide range of SMs including flavonoids (Gundlach *et al.*, 1992), putrescine (Horbowicz *et al.*, 2011), and benzoxazinoid derivatives (Oikawa *et al.*, 2002). That low [CO_2_] reduced SM synthesis via down-regulation of JA-dependent pathways is corroborated by evidence that deficiencies in potassium (Troufflard *et al.*, 2010) and phosphate (Khan *et al.*, 2016) can enhance SM production via up-regulation of JA and JA-Ile in Arabidopsis (Troufflard *et al.*, 2010; Khan *et al.*, 2016). In contrast, SA concentrations were higher in leaves at low than at ambient [CO_2_], indicating that low C availability reduces leaf SM production independent of the SA signalling pathway. Given that SA and JA are antagonistic (Zavala *et al.*, 2013; Sun *et al.*, 2016), it is possible that the up-regulation of SA signalling is associated with the suppression of JA signalling at low C availability.

In contrast, under elevated [CO_2_], leaf JA and SA levels changed independently from each other across developmental stages but both may nonetheless play a role in regulation of SMs. At 6L, leaf flavonoids and SA concentrations increased under elevated [CO_2_] but that was not the case for JA and JA-Ile, indicating that elevated [CO_2_] may stimulate short-term leaf flavonoid synthesis via SA (Zavala *et al.*, 2013; Sun *et al.*, 2016). At 8L, however, plants grown at elevated [CO_2_] had higher leaf JA and JA-Ile concentrations than at ambient [CO_2_] and probably induced flavonoid production. These dynamics mirror a potential long-term acclimation of photosynthesis at elevated CO_2_, as indicated by declines in RGR and soluble sugars in all tissues at elevated [CO_2_]. Deeper insights into such long-term leaf acclimation to elevated [CO_2_] via hormonal regulation require assessments of hormones and SMs over the entire plant developmental gradient.

In roots, however, while SMs and SA remained relatively constant, JA and JA-Ile concentrations were higher at elevated [CO_2_] than at ambient [CO_2_]. We suggest here that JA-dependent signalling for SM production in roots was probably constrained by N availability, given that root SMs are N-rich compounds and that assimilation of nitrate into organic N compounds may be limited under elevated [CO_2_] (Bloom *et al.*, 2010). Therefore, N availability, rather the JA- and JA-Ile-dependent signalling, determined the response of root SMs to elevated [CO_2_].

### Potential sugar signalling for IAA and JA synthesis

Soluble sugars are essential substrates for growth and metabolism (Hartmann and Trumbore, 2016), but also act as signalling molecules that interact with plant hormones to mediate plant stress responses (Rolland *et al.*, 2006; Lastdrager *et al.*, 2014). Our results revealed that accumulation of soluble sugars with increasing [CO_2_] positively correlated with weighted IAA and JA concentrations, allowing our experiment to demonstrate nicely the interaction of soluble sugars and IAA and JA at the whole-plant level. It has been shown that glucose (Sairanen *et al.*, 2012) and sucrose (Lilley *et al.*, 2012) can both stimulate IAA biosynthesis and growth rates. In contrast, jasmonates reduced glucose and fructose concentrations in *Nicotiana attenuata* leaves (Machado *et al.*, 2015) and play a synergetic role with sucrose (Loreti *et al.*, 2008) and glucose (Guo *et al.*, 2013) in anthocyanin and glucosinolate biosynthesis. Hence, it is more likely that accumulation of soluble sugars at high [CO_2_] stimulated whole-plant IAA and JA synthesis rather than vice versa. This sugar signalling pathway may also be a mechanism that triggers IAA and JA biosynthesis under cold, drought, and nutrient limitation, as these stresses decrease growth earlier than photosynthesis, thus allowing surplus carbon to be allocated to carbohydrates (Herms and Mattson, 1992; Palacio *et al.*, 2014). In contrast to IAA and JA, soluble sugars were not correlated with weighted ABA and SA concentrations, possibly because low [CO_2_] may trigger leaf ABA and SA biosynthesis via signalling pathways independent of sugar availability, for example ROS accumulation from excess excitation energy and photorespiration (Galvez-Valdivieso *et al.*, 2009; Herrera-Vásquez *et al.*, 2015).

Light intensities (8 mol m^−2^ d^−1^) were much lower in the greenhouse than usually occur in the field, and it might be that plants grown at ambient and elevated [CO_2_] were light limited. However, the general agreement of plant responses in our study with results from field experiments downplays the importance of light limitation. Moreover, higher light intensities in the field are likely to amplify CO_2_-induced responses, such as increases in assimilation, sugars, RGR, and secondary metabolites, as well as corresponding IAA and JA levels. Consequently, the suboptimal light conditions in the greenhouse underscore the robustness of our findings as the observed patterns are likely to be more pronounced in field-grown plants.

### Outlook

Our study is an initial step towards unravelling whole-plant hormonal regulation of growth and defence in response to changing [CO_2_] including low [CO_2_]. We hypothesize that low [CO_2_]-induced changes in ROS play important roles in leaf ABA and SA signalling, but this must be specifically addressed in future low [CO_2_] studies. Hormones are highly interactive (Peleg and Blumwald, 2011), and therefore interactions between hormones, such as JA and SA, will help to establish a conceptual framework for the complex hormonal regulatory mechanisms of plant response to changing [CO_2_] (Fig. 9).

Further progress in our understanding of hormonal whole-plant growth regulation can be achieved by combining measurements of bulk tissue hormone concentrations with investigations on hormone distribution and translocation across different tissues/organs. For example, root growth and development is regulated by auxin that has been synthesized in the shoot apex and transported to root tips to result in local auxin concentration peaks (Peer *et al.*, 2011). Similarly, ABA is stored mainly in the mesophyll cell, but a recent study shows that only ABA concentrations in guard cells control stomatal responses to elevated [CO_2_] (Chater *et al.*, 2015). Experiments with mutants will provide more direct evidence for causality in hormonal regulation, and future studies at the transcriptional level should focus on biosynthetic and, in particular, responsive genes involved in hormonal signalling pathways. For example, soluble sugars may stimulate IAA via the phytochrome-interacting factor (PIF) family of transcription factors (Lilley *et al.*, 2012; Sairanen *et al.*, 2012). Auxin-responsive *GH3* (Du *et al.*, 2012, 2013) and *Aux/IAA* genes (Jung *et al.*, 2015), and the JA-responsive jasmonate ZIM-domain (JAZ) gene family (Du *et al.*, 2013; Riemann *et al.*, 2015) are signalling repressors and have been shown to be involved in drought and cold tolerance.
